# Clinical studies on core-carrier obturation: a systematic review and meta-analysis

**DOI:** 10.1186/s12903-017-0459-1

**Published:** 2017-12-29

**Authors:** Amy Wai-Yee Wong, Shinan Zhang, Samantha Kar-Yan Li, Chengfei Zhang, Chun-Hung Chu

**Affiliations:** 1 Faculty of Dentistry, The University of Hong Kong, Hong Kong, SAR China; 2 School of Stomatology, Kunming Medical University, Yunnan, China; 3 3B53A, Prince Philip Dental Hospital, 34 Hospital Road, Hong Kong, SAR China

**Keywords:** Thermafil, Core carrier, Obturation, Endodontics, Systematic review, Meta-analysis

## Abstract

**Background:**

This systematic review aimed to evaluate the clinical performance of core-carrier obturation in endodontic treatment.

**Methods:**

Keywords of “(core carrier OR Thermafil) OR (cold lateral condensation OR lateral condensation) OR (warm vertical condensation OR vertical condensation) AND (obturation OR root canal filling) AND clinical study” were searched for all obtainable publications up to year 2017 in the databases of PubMed, ScienceDirect, EMBASE, Scopus and Web of Science. The success rate, short-term postoperative pain, overfilling and adaptation of core-carrier obturation from clinical studies were selected. Reviews, laboratory studies, animal studies and irrelevant reports were excluded.

**Results:**

1349 relevant articles were identified with 149 duplicated articles removed and 1173 irrelevant articles were excluded after screening. The titles and abstracts of the 19 identified articles were screened in the systematic review. The full texts of remaining articles were retrieved with data extracted for meta-analysis on the success rate, postoperative pain, overfilling and adaptation of obturation. The pooled success rate of core-carrier obturation was 83% (95% CI: 69%-91%). The pooled incidence of 1-day and 7-day short-term postoperative pain were 35% (95% CI: 15%-62%) and 6% (95% CI: 1-35%). The pooled proportion of teeth with overfilling and adequate adaptation of the obturation material were 31% (95% CI: 18%-50%) and 85% (95% CI: 75%-91%), respectively.

**Conclusions:**

The success rate of endodontic treatment using core-carrier obturation was 83%. Short-term postoperative pain was not uncommon (24%). Most teeth (85%) had adequate adaptation using core-carrier obturation material, but a considerable amount of teeth (31%) had overfilling.

## Background

The debridement and neutralization of any tissue, bacteria or inflammatory products within the root canal system is important for endodontic success. The outcome of endodontic treatments does not rely on a proper disinfection process only, but also on tight-sealed fillings of the canals as barriers to prevent re-infection. Therefore, root filling material is necessary to obturate the root canal in fluid tight seal 3-dimensionally on the main canal as well as the accessory canals. The ideal root filling material should have inert properties, good adhesive ability and result in voids-free obturation along the root canals. At present, the ideal root filling material is not available.

Since the introduction by Bowman in 1867, Gutta-percha has been the most commonly used solid core endodontic obturation material worldwide [1]. The root canal was packed with this non-plastising gutta-percha in cold lateral compaction, which was gradually moved towards a thermoplastising rubber-like material aimed at increasing root canal adaptability [2]. The cold lateral condensation technique is the most frequently used obturation techniques by general dentists, and it is used in many countries, such as Belgium [3], Hong Kong [4], India [5], Iran [6], Jordan [7], Saudi Arabia [8, 9], Turkey [10], UK [11] and the USA [12]. One of the disadvantages of the cold lateral condensation technique is that gutta-percha cones do not adapt properly to canal walls, particularly in the presence of irregularities in the canal, such as presence of isthmus, C-shaped morphology, resorptive defect and accessory canals. Inadequate adaptation poses microleakage of fluid along the obturated root canals. Clinicians and researchers looked for alternative obturation methods were reported [13, 14]. Contemporary endodontic obturation includes thermoplasticised techniques, such as warm vertical condensation and core-carrier obturation. These obturation methods make use of heat to plasticise the gutta-percha for higher degree of homogeneity and better canal adaptation [2, 13, 15]. A survey in the USA reported that core-carrier obturation was the second most frequently used obturation method among general dentists [12].

The Thermafil obturator as a simple obturation method for endodontic treatment was introduced by Johnson in 1978 [16]. It was the first core-carrier obturation technique that used heated alpha-phase gutta-percha on a metal carrier prior to obturate the root canals. The materials of the core-carrier obturator continued to evolve from stainless steel, to titanium, plastic and crosslinked gutta-percha obturator. The number of clinicians, in particular general dentists, who favoured the use of core-carrier obturator was increasing [12, 17]. This study was a systematic review to evaluate clinical success rate, short-term postoperative pain, overfilling and adaptation of the obturation material using core-carrier obturation techniques in endodontic treatment.

## Methods

### Literature search

A literature search was conducted to find descriptions using the 5 databases, which were MEDLINE database (PubMed), ScienceDirect, Excerpta Medica Database (EMBASE), Scopus and Web of Science. The keywords “(core carrier OR Thermafil) OR (cold lateral condensation OR lateral condensation) OR (warm vertical condensation OR vertical condensation) AND (obturation OR root canal filling) AND clinical study” were used to search for all obtainable publications up to December 2017. Two authors of this study performed the literature search independently. They screened the titles and abstracts of the identified articles. Duplicate articles, reviews, laboratory studies, animal studies and irrelevant reports were excluded. The remaining articles were retrieved with full texts, which were assessed for the relevance to this systematic review. The references of all the articles were checked to identify additional pertinent articles. Data extraction and analysis were performed and reviewed. Any disagreements on study inclusion, data extraction and analysis were discussed with the third author, until consensus was reached. The study design of the selected studies were evaluated on their risks of bias according to the Cochrane Handbook for Systematic Reviews of Interventions (The Cochrane Collaboration Version 5.1.0) [18].

### Study selection

The assessment variables for clinical studies of endodontic treatment included the treatment success, short-term postoperative pain, apical extrusion (overfilling) and quality (adaptation) of the root canal filling. Studies reporting core-carrier obturation alone or by comparison with other obturation methods were included in this review. The treatment success in this review was defined as both clinical success and radiographic success. The clinical success was the treated tooth without symptoms of tenderness towards percussion, pain sensation, abscess and any endodontic-related symptoms. Radiographic success was resulted from absence of periapical radiolucency in intraoral radiographs. The short-term postoperative pain was defined as the pain encountered within 1 week from the time of obturation. In this study, we reported the postoperative pain in 1 day and after 7 days separately based on the results of selected studies. The overfilling of the obturation material beyond the radiographic apex was evaluated. The adaptation of the obturation material was regarded as adequate when it was uniformly filled without visible voids or canal spaces in radiographic assessment. There was no consensus in reporting the time used for obturation in the studies and a summary was performed without statistical analysis [19, 20].

### Statistical analysis

The four assessment variables including treatment success, short-term postoperative pain, overfilling and adaptation of obturation materials in endodontic treatment were extracted from each included study. Data were retrieved from tables, figures and the main text of the articles.

The pooled overall prevalence in the four assessment variables (pooled success rate, pooled incidence of 1 day and 7 days short-term postoperative pain, pooled overfilling proportion and pooled proportion of adequate adaptation of the obturation material), separated meta analyses using logistic-normal random effect model [21] were performed by the Stata procedure metaprop_one [22]. The weighting in the proportion estimation was not explicit because parameter estimation was an iterative procedure.

Although this review primarily aimed to evaluate the clinical performance of core-carrier obturation in endodontic treatment, most studies used cold lateral condensation to compare core-carrier obturation. Thus, a direct comparison of the clinical performance of core-carrier obturation with cold lateral condensation was also performed in this review. The pooled relative risk (RR) in the four assessment variables were analysed using meta-analysis with DerSimonian and Laird random effects method [23] by the Stata procedure metan [24] using the cold lateral condensation technique as the control group. In addition, comparison of the clinical performance of core-carrier obturation with other common obturation methods was conducted. Meta-analysis using logistic-normal random effect model for each common obturation method in success rate and postoperative pain was performed. Heterogeneity tests were performed for each meta-analysis for the reference. The Stata 13.1 software (StataCorp LP, College Station, TX, USA) was employed in the statistical analysis. The results were presented in forest plots and the tests were set as two-tailed tests with the 0.05 significance level.

## Results

**Fig. 1:**
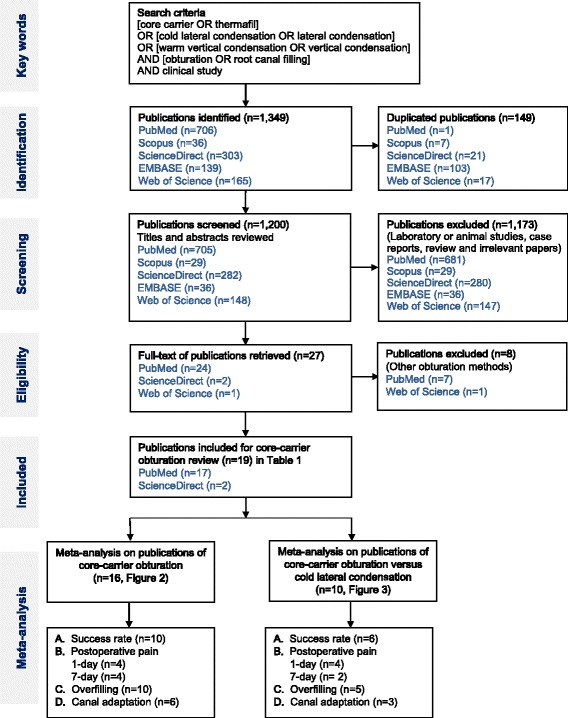
Flowchart of the literature search

The search identified 1349 potentially relevant articles in the 5 databases; 149 duplicated articles were removed. The titles and abstracts of 1200 publications were screened. After screening, 1173 papers were excluded because they were laboratory or animal studies, review papers, case reports, data studies or irrelevant reports. Eight clinical studies of irrelevant obturation methods were excluded. The remaining 19 publications of core-carrier obturation with full texts were retrieved. A manual search was performed on the references of these 19 papers and no additional reference was found. Therefore, 19 publications were included in this systemic review (Fig. 1). They were evaluated for their methodology and risk of bias (Table 1). Among these 19 studies, 11 papers reported the treatment success [1, 15, 19, 20, 25–31], eight papers reported short-term (within 7 days) postoperative pain [17, 19, 25, 27, 28, 32–34], 11 papers reported overfilling [15, 17, 19, 20, 25, 27, 30, 35–38] and seven papers reported the adequate adaptation of root canal filling [15, 20, 25, 30, 35, 37, 38].

**Table 1:**
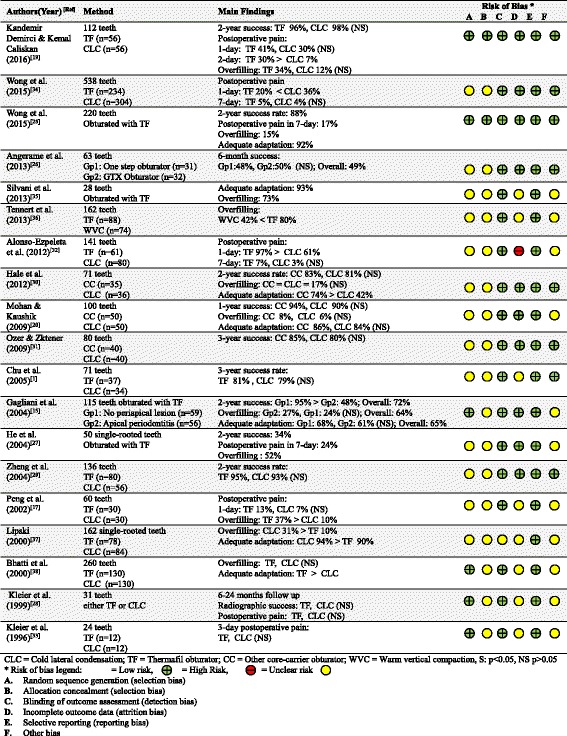
Summary of clinical studies on core-carrier obturation

**Fig. 2:**
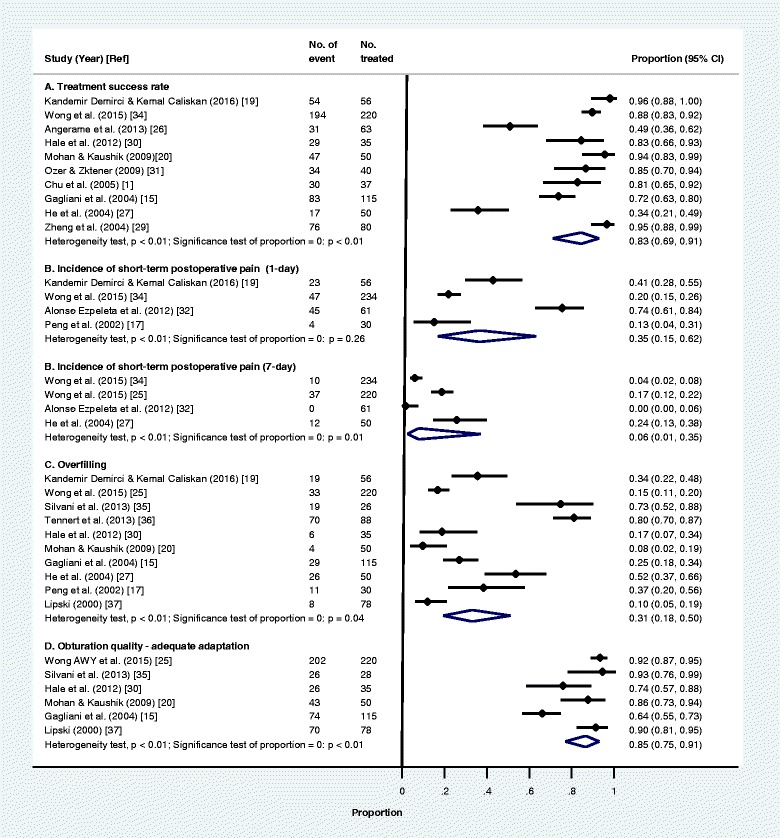
Meta-analysis of core-carrier obturation studies on treatment success, incidence of short-term postoperative pain, overfilling and obturation quality

The pooled proportion of treatment success, incidence of short-term postoperative pain, overfilling and obturation quality (adequate adaptation) in teeth obturated with core-carrier obturators, were summarised in Fig. 2. 10 of the 11 papers reporting the treatment success reported the exact success rate or the exact number of success cases for further meta-analysis. The pooled success rate of core-carrier obturation was 83% (95% confidence interval (CI): 69%-91%; *p* < 0.01) which was significantly different from zero. Seven of the eight studies reporting the exact 1 day and 7 days postoperative pain rate or the exact number of postoperative pain cases for further meta-analysis. The pooled incidence of 1 day and 7 days short-term postoperative pain were 35% (95% CI: 15%-62%; *p* = 0.26) and 6% (95% CI: 1-35%; *p* = 0.01) respectively which was not statistical significantly different from zero. The overall short-term postoperative collected within 1 week was 24% (95% CI: 15%-36%; *p* < 0.01).

Ten of the 11 studies reporting overfilling reported the exact overfilling rate or the exact number of overfilling cases and six of the seven studies reporting adaptation of the obturation reported the exact adaptation rate or the exact number of adaptation cases for further meta-analyses. The pooled proportion of teeth with overfilling and adequate adaptation of the obturation material were 31% (95% CI: 18%-50%; *p* = 0.04) and 85% (95% CI: 75%-91%; *p* < 0.01), respectively (Fig. 2).

**Fig. 3:**
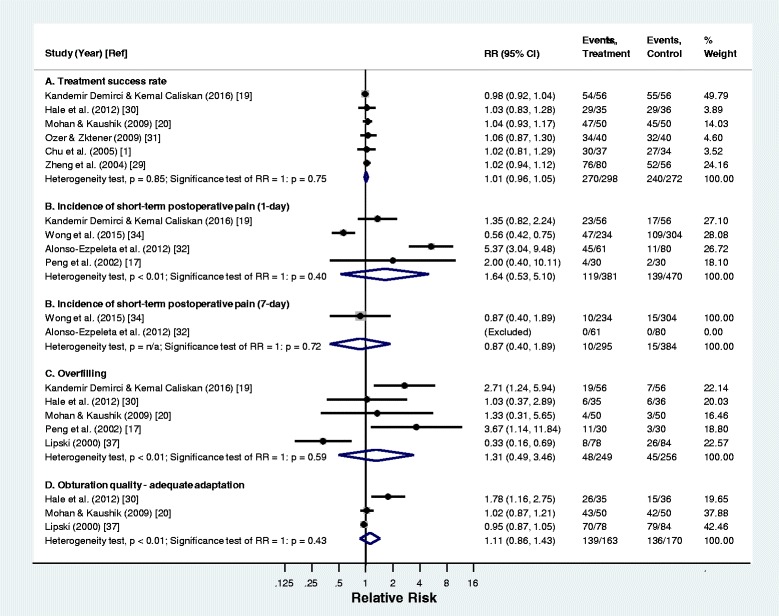
Forest plots of core-carrier obturation versus cold lateral condensation according to treatment success, incidence of short-term postoperative pain, overfilling and obturation quality

The results of meta-analysis of the treatment success rate of core-carrier obturation versus cold lateral condensation extracted from the six selected studies [1, 19, 20, 29–31] were presented in forest plots in Fig. 3. Studies reporting the core-carrier obturation alone were excluded from this analysis. The forest plot showed no significant difference in treatment success between core-carrier obturation and cold lateral condensation (RR = 1.01 with 95% CI: 0.96–1.05; *p* = 0.75). Results of meta-analysis on two studies using warm vertical compaction [39, 40] found the treatment success rate was 84% (95% CI: 77%-89). This treatment success rate was not significantly different from that of core-carrier obturation. Meta-analysis on four selected studies [17, 19, 32, 34] showed that the incidence of 1 day short-term postoperative pain (RR = 1.64 with 95% CI: 0.53–5.10; *p* = 0.40) and two selected studies [32, 34] of 7 days postoperative pain (RR = 0.87 with 95% CI: 0.40–1.89; *p* = 0.72) of core-carrier obturation were also not significantly different from that of cold lateral condensation. Likewise, the forest plot did not show significant differences in the overfilling (RR = 1.31 with 95% CI: 0.49–3.46; *p* = 0.59) and adequate adaptation (RR = 1.11 with 95% CI: 0.86-1.43; *p* = 0.43) between core-carrier obturation and cold lateral condensation of the selected studies (Fig. 3).

Three papers had reported the treatment or obturation time they all found that the time required was significantly shorter using core-carrier obturation than cold lateral condensation [17, 20, 41]. Only one study [20] reported the comparison of mean obturation time between core-carrier obturation and cold lateral condensation. This study reported that the core-carrier obturation times were 21 min for multiple canals and 13 min for a single canal, whereas the obturation times for lateral condensation were 28 min for multiple canals and 17 min for a single canal [20].

## Discussion

The core-carrier obturation technique was getting more popular in endodontic treatment, in particular for general practitioners [12]. Laboratory studies of core-carrier obturation were numerous; however, they were performed using extracted teeth, mimicked tooth models and plastic blocks, which were different from clinical settings. A systematic review for clinical studies on core-carrier obturation was therefore necessary but was not found in literature. In this study, five common databases, including PubMed, ScienceDirect, EMBASE, Scopus and Web of Science databases, were used for literature search. Although there are 19 clinical studies on core-carrier obturation, the number of teeth assessed in this study varied from 24 to 538. There are 8 studies with the number of teeth assessed less than 100. Among the 19 selected clinical studies, the detection bias and reporting bias are generally low. Some of the clinical studies on core-carrier obturation found in the databases had no details on how they randomised their samples. The “unclear” risk of bias on sample generation of randomisation revealed the need of better quality randomised clinical trials in this field. The initial aim of this review is to study exploratory into the performance of the core-carrier obturation. However, studies comparing core-carrier obturation with other obturation techniques, predominantly cold lateral condensation technique, were identified. Therefore, this review also compared the clinical outcome of core-carrier obturation with cold lateral obturation technique. It is noteworthy that the number of the studies was small. More studies are required to study the clinical outcomes including the success rate, incidence of postoperative pain, overfilling and quality of obturation.

Some studies evaluated the outcome of endodontic treatment based on radiograph [1, 20, 25, 26]. Radiograph was a 2-diemensional representation and it had limitations for evaluation. It was suggested that 3-dimensional radiographic methods increased the diagnostic value on treatment outcome [42, 43]. However the radiation dose was higher and need specialised equipment which may not be widely used in research purposes. The periapical radiograph method used was generally accepted by clinicians to assess healing progress and quality of obturation. In the radiographic assessment of the selected studies, the observers were independent and were blinded in the treatment method [1, 15, 26, 28, 36, 38]. For the assessment of postoperative pain after endodontic treatment, visual analogue scale [32] or likert scale [33] were used for grading the discomfort experienced by patients. These were reliable methods used for assessment of pain for dental procedures [32].

Among all the independent variables, the most important assessment for clinical protocol by operators was the success rate. In this systematic review, the success rate of endodontic treatment using core-carrier obturation and using cold lateral condensation were not statistically significant. In this study, the success rates of warm vertical compaction and core-carrier obturation were not statistically significant. The core-carrier obturation could be a reasonable alternative to conventional technique without compromising the treatment outcome. However, only six studies were included in this analysis; the sample size and power of this analysis were limited. Notwithstanding the similar treatment outcome between the two methods, the microleakage of obturation was hard to evaluate in clinical studies but regarded as an important factor influencing the treatment outcome. Studies reported that core-carrier obturators produced higher gutta-percha/sealer ratio, thus reducing apical leakage, and less cytotoxic by-products disintegrated from sealer than in cold lateral condensation [44, 45]. Laboratory studies showed that no significant difference was found in apical leakage between core-carrier obturation and cold lateral condensation [46–48].

Another important aspect in assessing clinical protocol was postoperative pain which was a key factor affecting patient satisfaction [49]. The results of this review found no significant difference in the postoperative pain of core-carrier obturation and cold lateral condensation. The pooled result of short-term postoperative pain in 1 day and 7 days obturated with a core-carrier obturator were 35% and 6% respectively, which was comparable with that of cold lateral condensation with 6% severe pain to 54% mild post-obturation pain [50]. Extrusion of the obturation materials beyond the root apex could be a reason for the pain and discomfort [19].

The adequate adaptation of core-carrier obturation compared with cold lateral condensation based on the two clinical studies could not demonstrate significant difference [20, 37]. The method adopted was 2-dimentional radiographic assessment, which was inferior to the 3-dimentional assessment with cone beam computed tomography. It was plausible that the voids created by cold lateral condensation during packing of gutta-percha with spaces left behind by spreader or shrinkage of sealer could increase the microleakage and thus affected the treatment outcome. A study reported that core-carrier obturation had less sealer and more gutta-percha and facilitated adaptation of the filling material along the root canal spaces [44]. A recent study reported that obturation by crosslinked gutta-percha core obturator consistently produced homogeneous obturation with lower incidences of voids compared with cold lateral condensation [51]. There was another study demonstrated the improvement on retrievability in endodontic re-treatment by crosslinked gutta-percha obturator than plastic core one [52]. Some clinicians suggested that core-carrier obturation enabled gutta-percha tag formation inside the dentinal tubules, especially when the smear layer was removed by combined irrigations [53, 54]. There were significantly greater wedging forces on obturation with conventional cold lateral condensation than with core-carrier obturation. Dentists tended to exert forces to the spreader during obturation so as to increase the adaptation of the cold lateral condensation. This should be avoided because this act increased the risk of tooth fracture. Core-carrier obturation might induce less vertical forces on the root canal and thus reduced the chance of root fracture after obturation. Therefore teeth with weakened remaining tooth structure or in doubtful prognoses, such as cracked teeth, might be better having core-carrier obturation than cold lateral condensation.

A drawback of the core-carrier obturator was less control of the root canal filling, which should be confined within a root canal space [55]. The overfilling after obturation with the core-carrier was greater as compared with cold lateral condensation [17, 20], while one study reported the contrary [37]. The contradictory result of this report study [37] did not explain the reason of cold lateral condition showed more overfilling than Thermafil over radiographic evaluation. Extrusion of gutta-percha or sealer might be influenced by a host’s periapical tissues, apical patency, canal tapering and a patient response to the pain sensation [55], and some of these factors could not be evaluated with an in vitro study. The clinical implications of overfilling might induce undesirable pain and possible pooling of sealer in the apical portion of the canal. The risk of thermal trauma and extrusion trauma were two important issues for a clinician to consider when using thermoplasticised gutta-percha. A laboratory study found that the temperature rise was below the critical level that caused biological breakdown to periodontal attachment [56]. A laboratory study demonstrated that the likelihood of overfilling was associated with the canal tapering [55]. A study reported that the risk of overfilling could be reduced by using a small amount of sealer and obturating the canal with the master cone that correlated with the last file size [46]. The use of contemporary instrumentation instruments might allow better control of the core-carrier obturation within designated working length, and further studies should be performed.

It is generally accepted by clinicians that the treatment or obturation time required was significantly shorter using core-carrier obturation than cold lateral condensation [17, 20, 25]. However, the factors affecting the treatment time are many. Operator skills and experience and complexity of the root canals system are two other important factors affecting the time for endodontic treatment [20]. A clinical study reported that the time used for core-carrier obturation was shorter than that for cold lateral condensation [19]. There were laboratory studies that reported similar results with used core-carrier obturation [17, 20, 57]. The obturation time was not a variable related to the outcome of endodontic treatment, and thus was not reported in this study. Nevertheless, it could be an important factor affecting dentists’ choice of obturation. Core-carrier obturation was a simpler thermoplastised technique than warm vertical condensation for mastering the skill. General dentists were generally satisfied and preferred to use core-carrier obturation because the chairside time can be reduced [12, 25].

Endodontic treatment is a common dental treatment to save teeth from extraction. The success rate of endodontic treatment was generally high compared to dental implants [58, 59]. The long term survival rate of compromised teeth that were endodontically treated was reported to be as high as 83% to 98% [60]. The advance in materials and instruments had changed significantly regarding the protocols of endodontic treatment in recent decades. The use of thermoplasticised obturation could be an alternative to the traditional cold lateral condensation. It was easy and quick to master the skills of the core-carrier obturation technique. However, overfilling could be a concern. More clinical trials on core-carrier obturation using updated materials and instrument were needed.

## Conclusions

This systematic review found the success rate of endodontic treatment using core-carrier obturation was 83%. Short-term postoperative pain was not uncommon (24%). Most teeth (85%) had adequate adaptation using core-carrier obturation material, but a considerable amount of teeth (31%) had overfilling.

## Abbreviations

CC: Other core-carrier obturator
CI: Confidence interval
CLC: Cold lateral condensation
EMBASE: Excerpta medica database
MEDLINE: Medical literature analysis and retrieval system online / U.S. National Library of Medicine’s life science database
NS: Not significant
RR: Relative risk
S: Significant
TF: Thermafil obturator
WVC: Warm vertical compaction
